# Effects of timing of umbilical cord clamping on preventing early infancy anemia in low-risk Japanese term infants with planned breastfeeding: a randomized controlled trial

**DOI:** 10.1186/s40748-021-00125-7

**Published:** 2021-01-19

**Authors:** Eriko Shinohara, Yaeko Kataoka, Yukari Yaju

**Affiliations:** 1 Department of Nursing, School of Medicine, Yokohama City University, 3-9 Fukuura, Kanazawa-ku, Yokohama, Kanagawa 236-0004 Japan; 2 Division of Women’s Health and Midwifery, Graduate School of Nursing Science, St. Luke’s International University, 10-1 Akashi-cho, Chuo-ku, Tokyo, 104-0044 Japan; 3 Division of Epidemiology and Statistics, Graduate School of Nursing Science, St. Luke’s International University, 10-1 Akashi-cho, Chuo-ku, Tokyo, 104-0044 Japan

**Keywords:** Umbilical cord, Anemia, Jaundice, Breastfeeding, Randomized controlled trial

## Abstract

**Background:**

Japanese infants have relatively higher risk of anemia and neonatal jaundice. This study aimed to assess the effects of delayed cord clamping (DCC) on the incidence of anemia during early infancy in low-risk Japanese term infants with planned exclusive breastfeeding for 4 months. This study also aimed to explore the effects of DCC on neonatal jaundice.

**Methods:**

We conducted an open-label, parallel-arm, multicenter randomized controlled trial of DCC (clamping the cord after more than a minute or pulsation stops) vs. early cord clamping (ECC; clamping the cord within 15 s) at one birth center and two clinics in Japan. Low-risk pregnant women planning to have a vaginal birth and to exclusively breastfeed and term singleton infants delivered in cephalic presentation were included in this study. The primary outcome was spectrophotometric estimation of hemoglobin at 4 months. Secondary outcomes were anemia incidence at 4 months, four outcomes related to neonatal jaundice, hematocrit levels, and related outcomes.

**Results:**

Overall, 150 pregnant women were recruited. Participants (*N* = 138) were randomly allocated to two groups (DCC *n* = 68, ECC *n* = 70). There were no significant differences between the two groups in spectrophotometric estimation of hemoglobin at 4 months: mean difference = 0.1 g/dL, 95% confidence interval − 0.14, 0.35, DCC 12.4 g/dL, ECC 12.3 g/dL. Only the hematocrit levels on days 3 to 5 were significantly higher in the DCC group than in the ECC group: DCC 57.0%, ECC 52.6%, mean difference = 4.4, 95% confidence interval 2.61, 6.20. There were no significant differences in other secondary outcomes, including outcomes related to neonatal jaundice.

**Conclusion:**

Among low-risk Japanese term infants with planned exclusive breastfeeding, DCC showed no significant effects on spectrophotometric hemoglobin levels at 4 months compared with ECC. We observed significantly higher hematocrit levels on days 3 to 5 in infants who underwent DCC, while these levels were within the normal range. Jaundice outcomes remained similar to those of infants who underwent ECC. Although a larger sample size is required to assess the effects of cord clamping on neonatal jaundice, DCC may prevent anemia in newborn infants.

**Trial registration:**

UMIN-CTR; UMIN000022573, 06/01/2016 - retrospectively registered, https://upload.umin.ac.jp/cgi-open-bin/ctr/ctr_view.cgi?recptno=R000023056

## Background

Exclusive breastfeeding is one of several risk factors for anemia in infancy [1]. In Japan, over half (54%) of mothers exclusively breastfeed for at least 3 months after birth and over one-third continue to exclusively breastfeed at 6 months [2]. Although there is no reported nationwide survey on anemia in infancy, a prefectural report from Okinawa concluded that 10–14% of breastfed infants have anemia; a higher rate of anemia than that among infants given formula [3]. Another study reported that exclusive breastfeeding was associated with a higher incidence of anemia in infancy compared to that with partial breastfeeding and formula [4].

Several randomized controlled trials have been conducted to clarify the effects of delayed cord clamping (DCC) on the prevention of anemia in infancy. In a study in Sweden, ferritin levels at 4 months were significantly higher with DCC than that with early cord clamping (ECC) [5]. Similarly, a study on Mexican infants found that ferritin and total body iron were significantly higher in the DCC group at 6 months [6]. In East Asia, a study from China reported that serum ferritin levels were significantly higher in the DCC group at 4 months [7]. Subgroup analysis in the study in Mexico showed that DCC improved body iron and stored iron, in neonates at high risk of anemia who were exclusively breastfed [6]. Therefore, DCC may be considered a useful intervention among Japanese exclusively breastfed infants. However, DCC may increase the risk of neonatal jaundice. In the Swedish study, there was no significant difference between DCC and ECC groups for neonates treated with phototherapy [5]. Similarly, there were no significant differences for clinical jaundice between the two groups in the Mexican study [6]. In East Asia, Chinese and Taiwanese studies reported no significant effects on jaundice-related outcomes of total serum bilirubin (TsB) [7, 8]; however, a Cochrane systematic review including unpublished data concluded that DCC may increase the risk of neonates requiring phototherapy [9]. Although previous studies have reported the benefits of DCC in preventing anemia in infancy [5–7], East Asians have a relatively higher risk of neonatal jaundice [10] and DCC should be performed with caution in this at-risk population. In Japan, as neonates are at high risk of hyperbilirubinemia [11], DCC has not yet been recommended due to concerns that DCC increases the risk of developing hyperbilirubinemia in neonates [12]. In Japan, approximately 90% of clinics or hospitals have adopted a policy of ECC, conversely nearly 70% of midwifery birth centers have adopted a policy of DCC [13]. A retrospective cohort survey reported that only 1.8% of neonates required phototherapy at a birth center where DCC was conducted [14]. In addition, a recent observational study reported no significant association between the timing of umbilical cord clamping, infant anemia at 3 to 5 months, and neonatal jaundice [15]. While there is a clear need for high-quality research in order to make the appropriate recommendation for the timing of umbilical cord clamping [12], there have been no randomized controlled trials on the timing of cord clamping in a Japanese population. A trial comparing DCC and ECC may also generate evidence applicable to other countries. Furthermore, updating evidence-based reviews is important for clinical decision-making. The most-recent Cochrane systematic review on the timing of cord clamping for term infants was updated in 2013 [9]. In contrast, the Cochrane review on the timing of cord clamping for preterm infants was updated in 2019 and this found that DCC may reduce the risk of death among preterm infants before discharge [16]. Therefore, it is important to also update the review for term infants, and the results of this study may contribute to this process by providing new clinical trial information.

Therefore, the main purpose of this study was to assess the effects of DCC on the incidence of anemia in low-risk Japanese term infants, with planned exclusive breastfeeding, at 4 months. The secondary purpose was to assess whether DCC may increase the risk of neonatal jaundice.

## Participants, ethics, and methods

### Study design

This multicenter randomized controlled trial was conducted at two clinics and one birth center in Kanagawa, Japan between December 2015 and November 2016. The study protocol was approved by the Institutional Review Board of St. Luke’s International University and registered with UMIN-CTR in Japan (UMIN000022573; dated June 01, 2016 - retrospectively registered, https://upload.umin.ac.jp/cgi-open-bin/ctr/ctr_view.cgi?recptno=R000023056). This clinical trial adhered to the clinical research ethical guidelines for human subjects established on April 27, 2015 by the Ministry of Education, Culture, Sports, Science and Technology, and the Ministry of Health, Labor and Welfare, Japan.

### Participants

Participants were non-smoking pregnant Japanese women, planning a vaginal birth and exclusive breastfeeding, and neonates who were term, singleton, and in cephalic presentation.

Participants were excluded if they had any maternal complications, fetal complications, or emergency cesarean section, were transferred to another hospital during pregnancy or delivery, were not literate in Japanese, or were unable to return in 4 months.

All potential participants who met the inclusion criteria were selected from the medical record; both verbal and written research information were provided, as well as a consent form. After agreeing to participate in the study, participants signed the consent form. For the inclusion of neonates in the study, their guardians (mother or father) were provided with information regarding the study and then signed the consent form on the infants’ behalf.

### Randomization and masking

Randomization was performed centrally using the Mujinwari system (Iruka System Corporation [17], Tokyo, Japan) with a block size of four via access to the internet. Because of the different characteristics of participants in each facility, randomization was stratified by institution. E.S or research assistants performed allocation when normal progress leading to vaginal delivery was predicted (i.e. full dilation of the cervix in primiparas or 6–8 cm dilation of the cervix in multiparas). Allocations were provided to the midwives who were to perform the intervention, and assistant midwives, who measured the time from neonatal delivery to cord clamping, were informed on the group assignment of the participants at the time of delivery. Masking was not applied in this trial. Because of the characteristics of the intervention, participants and midwives in charge of the intervention were aware of treatment allocation. Research assistants and E. S, who evaluated the outcomes, were also aware of allocation.

### Intervention

Midwives clamped the cord using a Kocher clamp after more than a minute following neonatal delivery or when cord pulsation stopped in the intervention group and within 15 s after neonatal delivery in the control group. To ensure similarity in settings, all neonates were placed on the chest or abdomen of their mothers just after delivery. Moreover, the position of the mothers was set to about 30-degree semi-Fowler position after neonatal delivery.

Midwives involved in the deliveries were instructed on the intervention procedure using the study protocol and had been trained before the study commenced.

### Outcomes

The primary outcome was the spectrophotometric hemoglobin (SpHb) level at 4 months. Secondary outcomes were as follows: incidence of anemia (SpHb < 11.0 g/dL), TsB on days 3–5, incidence of over the excess value of TsB (High TsB value based on the nomogram by Imura [18]), incidence of phototherapy, transcutaneous bilirubin level on days 1–4, hematocrit on days 3–5, high hematocrit value (hematocrit level ≥ 65% by capillary blood), birth weight, infant vital signs (heart rate, respiratory rate, and temperature) after birth, infant growth (weight, height, head circumference, and chest circumference) at 1 and 4 months of age, adverse effects (seizures, admission to neonatal intensive care units, neonatal death), and maternal hemorrhage (third stage, within 2 h). Data were collected at the following time points: during hospital or clinic stay and at 1 and 4 months of age.

### Measurements

#### SpHb monitoring

Non-invasive and continuous Hb measurements using pulse oximeter (Radical-7®; Mashimo, Irvine, CA, USA) technology was conducted to determine the SpHb levels at 4 months of age. Non-invasive estimation of Hb levels by pulse-CO-oximetry in infants and neonates undergoing surgery showed a significant correlation with the invasive standard laboratory measurement of total Hb (r = 0.73, *p* < .001) and demonstrated clinically acceptable agreement with standard laboratory Hb measurements [19]. This continuous monitoring of SpHb in the stable state was required during measurement. The value was measured 2 min after a stable SpHb was detected. We choose SpHb as the primary outcome because it correlated with serum Hb and is a non-invasive method to collect data in neonates.

#### Anemia

If the SpHb level was less than 11.0 g/dL at 4 months, infants were defined as anemic.

#### TsB

Blood samples were collected to measure TsB concentration on days 3–5. To minimize the invasiveness of the procedure for neonates, blood samples for measuring TsB were collected during routine blood sampling for congenital metabolic disorder mass screening. Blood was collected using hematocrit capillary tubes by a midwife or nurse and immediately centrifuged; then, the TsB level was measured using a BL-300 jaundice meter® (TOITU, Tokyo, Japan) or similar device.

#### Over the excess value of total serum bilirubin

The bilirubin nomogram by Imura [18] for the prediction of hyperbilirubinemia that requires treatment was used because it is commonly used in the clinical setting in Japan. High TsB values based on the nomogram by Imura [18] were defined as “over the excess value of TsB”.

#### Phototherapy

The number of neonates who underwent phototherapy was counted.

#### Transcutaneous bilirubin

A non-invasive bilirubinometer (JM-103® or JM-105®; Konica Minolta, Tokyo, Japan) was used to measure transcutaneous bilirubin concentration by placing the device on the chest and forehead of the neonate on days 1–4. The value used was calculated as the average of one measurement each from the forehead and chest. Transcutaneous bilirubin shows a high correlation with TsB [20] and is routinely used for screening for hyperbilirubinemia in clinical settings.

#### Hematocrit

The blood samples collected for measuring TsB were also used to determine the hematocrit. The hematocrit was measured after centrifuging capillary blood.

#### High hematocrit value on days 3–5

If the hematocrit level was ≥65% by capillary blood, infants were classified as high hematocrit value.

### Data collection

Mothers and neonates were checked by midwives or nurses immediately after birth, at 1 h, and at 2 h and cared for according to routine practice. The data on maternal hemorrhage and neonatal vital signs were collected at those time points. In two facilities, mothers remained in hospital for 5 days, except those who were willing to be discharged earlier or required transfer to another hospital for the management of abnormalities. In one clinic, the routine stay was 3 days. In all facilities, the practice of rooming-in was followed, and breastfeeding was encouraged from soon after birth. Transcutaneous bilirubin levels in all neonates were checked using a bilirubinometer every morning. On day 4, blood collection for mass screening generally occurred. At that time, additional blood was collected for measuring TsB and hematocrits. However, blood tests were also performed on additional days in neonates with high transcutaneous bilirubin levels identified on daily checks. For the neonates undergoing multiple blood tests, the highest values of the TsB or hematocrit were used as outcome data. After discharge, infants usually had a check-up at 2 weeks to evaluate weight and jaundice, and mothers also received breastfeeding support from the midwives. At the 1-month check-up, the doctor or midwives assessed the neonates’ health including growth, jaundice, and nutrition (exclusive breastfeeding, mixed, or formula milk). One month’s data were collected at that point. At 4 months, the researcher collected growth, nutrition, and SpHb data. At a time, convenient for the mothers, E.S or a research assistant collected demographic characteristics of mothers and neonates and delivery outcomes.

### Sample size

This study explored the effects of cord clamping timing on Hb status at 4 months of age by comparing the DCC and ECC groups. From a previous cohort study [15], the estimated mean SpHb value in the ECC group was approximately 11.5 g/dL. Previous studies [21, 22] found that the Hb level was 11.5 g/dL in the ECC group and 12.0 g/dL in the DCC group, and an effect size of 0.5 g/dL was predicted. For the primary outcome measure of SpHb, based on 80% power to detect a significant difference of 0.5 g/dL with a 0.8 g/dL standard deviation (*α* = 0.05, two-sided), 40 participants were required for each study group. Assuming a dropout rate of 20%, a minimum total of 100 patients was required.

### Statistical analysis

Descriptive statistics were used to summarize the participants’ backgrounds. To compare the DCC and ECC groups, a *t*-test was used for the primary outcome of mean difference (MD) in SpHb. For secondary outcomes, *t*-testing was used for MDs, the Mann-Whitney *U* test was used for non-parametric variables, and the chi-squared test was used for bivariate analysis. Risk ratios (RR) and their confidence intervals (CI) were calculated, as well as confidence intervals for difference in means.

The primary outcome was assessed according to the intention-to-treat principle. One participant in the DCC group was not included in the analysis because data were missing due to technical difficulties. However, one subject in the DCC group who did not complete the protocol was included. Secondary outcomes were assessed using intention-to-treat principles for continuous variables and intention-to-treat for nominal variables. Per-protocol analysis and as-treated analysis were performed as adjunct methods. In per-protocol analysis, participants who deviated from the protocol were excluded from the analysis. In as-treated analysis, analysis was conducted as if participants were treated, regardless of allocation. All data were analyzed using IBM SPSS Statistics for Windows version 24.0 (IBM Corp., Armonk, NY).

## Results

### Baseline characteristics

**Fig. 1:**
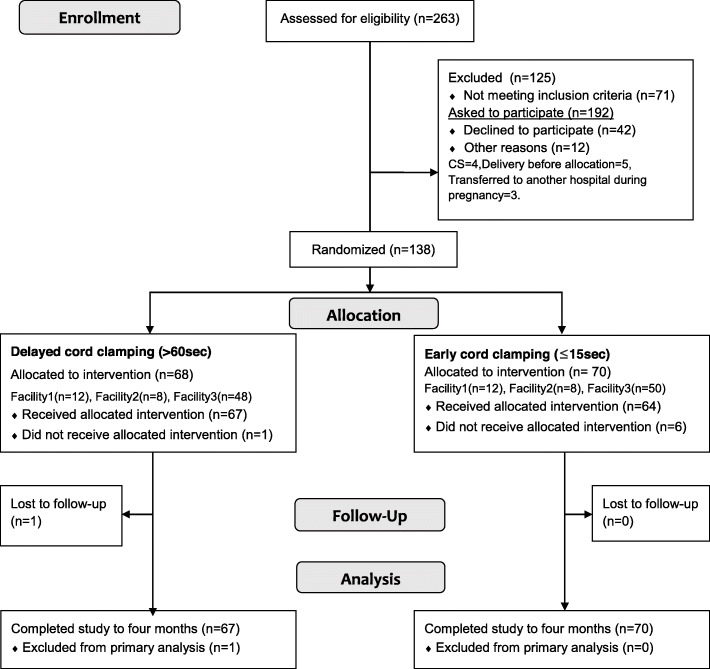
Flow diagram of the trial comparing timing of cord clamping, showing the number of participants followed up during the trial. a. Four (DCC *n* = 2/ ECC *n* = 2) were exclusively formula fed at 4 months

Between December 2015 and June 2016, a total of 263 women were screened for eligibility, and almost half (*n* = 125) were excluded after either not meeting the inclusion criteria (*n* = 71) or declining to participate in the study (*n* = 42) or not able to deliver at study facility (*n* = 12). A total of 150 women agreed to participate. Of these, 138 were randomly allocated to the DCC group (*n* = 68) or ECC group (*n* = 70). Figure 1 displays a flow diagram of trial recruitment and follow-up. At 4 months, only one infant in the DCC group had been lost to follow-up. Thus, in the final analysis, there were 67 infants in the DCC group and 70 in the ECC group. Some data for secondary outcomes and baseline characteristics were missing, but the number of missing values was small and considered to be missing at random, therefore imputation of missing values was not performed.

Baseline characteristics of mothers and neonates did not differ significantly between the two groups (Table 1). Median cord clamping time was 8 s (SD = 30 s, range 3–185 s) in the ECC group and 123 s (SD = 124 s, range 5–622 s) in the DCC group. One neonate in the DCC group underwent clamping before 60 s, and six neonates in the ECC group underwent clamping after 15 s (Fig. 1). Of note, 87.1% of infants in the DCC group and 80.6% of infants in the ECC group were exclusively breast fed and, in both groups, two infants were exclusively formula fed at 4 months (Fig. 1).

**Table 1:** Baseline Characteristics of Participants (*N* = 138)

	DCC (*n* = 68)		ECC (*n* = 70)	
**Maternal characteristics**				
Maternal age	30.5	(5.0)	31.1	(5.0)
Parity				
primipara	24	(35.3)	21	(30.0)
multipara	44	(64.7)	49	(70.0)
Anemia (Hb < 11.0 g/dl)	32	(47.1)	37	(52.9)
Blood type O (all Rh +)	17	(25.0)	16	(22.9)
History of photo therapy (only for multipara; *n* = 93,DCC = 44,ECC = 49)	7	(10.3)	7	(10.0)
**Neonatal characteristics**				
Gender				
male	34	(50.0)	36	(51.4)
female	34	(50.0)	34	(48.6)
Gestational age				
37w	8	(11.8)	0	(0.0)
38w	5	(7.4)	13	(18.6)
39w	34	(50.0)	26	(37.1)
40w	17	(25.0)	23	(32.9)
41w	4	(5.9)	8	(11.4)
Low birth weight (≧2500 g)	3	(4.4)	6	(8.6)
Maximum weight loss (%) (*n* = 134,DCC = 67,ECC = 67)	7.5	(1.8)	7.1	(1.7)
Meconium was not excreted at day 4 (*n* = 109, DCC = 54 ECC = 55)	25	(46.3)	28	(50.9)
Increased weight 0-4mths (g)	3603	(595.0)	3701	(685.1)
**Delivery**				
Type of delivery				
Duration of delivery	507	(372.1)	509	(381.7)
Instrumental delivery	3	(4.4)	0	(0)
Use of oxytocine (induction of labor)	4	(5.9)	7	(10.0)
Use of oxytocine immidiately after birth	57	(83.8)	54	(77.1)
Duration of third stage of labor (mins)	9.2	(5.9)	8.2	(5.2)
Placenta (g)	569	(80.4)	563	(85.7)
Apgar score (1 min) less than7	1	(1.5)	3	(4.3)
Apgar score (5 min) less than7	0	(0)	0	(0)
Cephelohematoma	0	(0)	2	(2.9)
**Nutrition**				
Type of nutrition (during confinement)				
exclusive breast feeding	40	(59.7)	48	(70.6)
mixed	27	(40.3)	20	(29.4)
formula milk	0	(0)	0	(0)
Type of nutrition (1 month)				
exclusive breast feeding	50	(74.6)	53	(75.7)
mixed	17	(25.4)	17	(24.3)
formula milk	0	(0)	0	(0)
Type of nutrition (4 month) (*n* = 137; DCC = 67, ECC = 70)				
exclusive breast feeding	54	(80.6)	61	(87.1)
mixed	11	(16.4)	7	(10.0)
formula milk	2	(3.0)	2	(2.9)
Weaning food (4 month)	0	(0)	0	(0)

Data are presented as mean (*SD*) or *n* (%)

### Primary and secondary outcomes

SpHb levels at 4 months were 12.4 g/dL in the DCC group and 12.3 g/dL in the ECC group, with no significant differences between the intervention and control groups (MD 0.1, 95% CI -0.14-0.35) (Table 2). Only one infant in the DCC group had a SpHb level lower than 11.0 g/dL and was screened as anemic (Table 2).

**Table 2:** Primary and Secondary Outcomes Analyzed by Intent to Treat

	DCC		ECC		Relative risk (95% CI) / Mean difference (95% CI)		*p* value
**Primary outcome**							
**Spectrophotometric hemoglobin at 4 months** (*n* = 137/DCC = 67,ECC = 70)	12.4	(0.8)	12.3	(0.6)	0.1	[−0.14, 0.35]	0.40
**Secondary outcome**							
**Neonates**							
1) **Anemia (SpHb < 11.0 g/dl)** (*n* = 137/DCC = 67, ECC = 70)	1	(0.7)	0	(0)	0.9	[0.64, 1.25]	0.49
2) **Total serum bilirubin (TsB) (days 3-5) (mg/dl)** ^a^ (*n* = 132/DCC = 66, ECC = 66)	12.8	(3.4)	12.2	(3.6)	0.6	[−0.61, 1.80]	0.33
3) **Over the excess level of TsB (%)** ^b^ (*n* = 132/DCC = 66, ECC = 66)	6	(9.1)	5	(7.6)	1.2	[0.39, 3.74]	1.00
4) **Phototherapy (%)** ^c^ (*n* = 133/DCC = 66, ECC = 67)	7	(10.3)	5	(7.1)	1.4	[0.48, 4.25]	0.37
5) **TcB level (days 1-4) (mg/dl)**							
day1 (*n* = 137/DCC = 68, ECC = 69)	5.0	(1.8)	4.6	(1.9)	0.3	[−0.29, 0.95]	0.29
day2 (*n* = 135/DCC = 67, ECC = 68)	8.7	(2.2)	8.6	(2.2)	0.1	[−0.63, 0.87]	0.75
day3 (*n* = 126/DCC = 62, ECC = 64)	11.4	(2.6)	10.9	(2.9)	0.5	[−0.45, 1.50]	0.29
day4 (*n* = 119/DCC = 58, ECC = 61)	11.9	(2.7)	11.4	(3.1)	0.5	[−0.57, 1.53]	0.37
6) **Hematocrit level (days 3-5) (%)** ^a^ (*n* = 132/DCC = 66, ECC = 66)	57.0	(5.2)	52.6	(5.2)	4.4	[2.61, 6.20]	< 0.01*
7) **High hematocrit (Hct≧65%)**	3	(4.6)	2	(3.0)	1.5	[0.26, 8.82]	0.68
8) **Birth weight (g)**	3118	(332.5)	3030	(354.5)	88.5	[−27.26, 204.19]	0.13
9) **Vital signs after birth**							
After birth							
heart rate (times/mins) (*n* = 131/DCC = 64, ECC = 67)	154	(15.3)	150	(13.2)	3.9	[−1.05, 8.83]	0.12
respiration (times/mins) (*n* = 129/DCC = 63, ECC = 66)	59.3	(9.5)	61	(11.6)	−1.7	[−5.45, 1.96]	0.35
body temperature (°C) (*n* = 129/DCC = 63, ECC = 66)	37.0	(0.5)	37.0	(0.5)	0.0	[−0.14, 0.20]	0.75
SpO2 (%) (*n* = 132/DCC = 65, ECC = 67)	98	(1.8)	98	(2.0)	−0.3	[−0.93, 0.37]	0.40
1 h							
heart rate (times/mins)	150	(10.4)	150	(11.8)	0.3	[−3.42, 4.09]	0.86
respiration (times/mins) *n* = 137/DCC = 68, ECC = 69)	56	(11.6)	57	(9.1)	0.5	[−4.61, 2.40]	0.54
body temperature (°C) (*n* = 137/DCC = 68, ECC = 69)	37.3	(0.5)	37.2	(0.5)	0.0	[−0.14, 0.22]	0.67
SpO2 (%)	99	(1.2)	99	(1.2)	−0.1	[− 0.48, 0.32]	0.70
2 h							
heart rate (times/mins) (*n* = 137/DCC = 67, ECC = 70)	142	(13.3)	140	(12.9)	1.6	[−2.85, 6.00]	0.48
respiration (times/mins) (*n* = 137/DCC = 67, ECC = 70)	52	(7.3)	52	(8.9)	−0.3	[−3.09, 2.44]	0.82
body temperature (°C) (*n* = 137/DCC = 67, ECC = 70)	37.2	(0.4)	37.2	(0.4)	0.0	[−0.14, 0.15]	0.93
SpO2 (%) (*n* = 137/DCC = 67, ECC = 70)	99	(1.1)	99	(1.5)	0.1	[−0.35, 0.54]	0.68
10) **Infant growth**							
1 month weight (g) (*n* = 136/DCC = 67, ECC = 69)	4206	(517.2)	4175	(421.4)	30.1	[−129.64, 189.93]	0.71
4 month weight (g) (*n* = 137/DCC = 67, ECC = 70)	6727	(751.1)	6731	(759.7)	−3.3	[−258.62, 252.09]	0.98
11) **Adverse effects**							
seizure (%)	0	–	0	–	–	–	–
admitted to NICU within 24 h (%)	0	–	0	–	–	–	–
neonatal death (%)	0	–	0	–	–	–	–
**Mother**							
12) **Haemorrhage**							
at third stage of labor (g)	288	(224.3)	275	(185.0)	13.3	[−55.84, 82.41]	0.71
total (until 2 h)	419	(283)	380	(194)	39.0	[−42.43, 120.26]	0.24

Data are presented as mean (*SD*) or *n* (%)

^a^ The highest value among blood sampling conducted during days 3-5 were used

^b^ Over the excess value are based on Standard of Imura (1985)

^c^ Two (DCC = 1, ECC = 1) received phototherapy before meeting the standard of phototherapy for prevention. One (ECC = 1) did not received phototherapy even over the level for the standard of phototherapy

*p < .05

Four markers of neonatal jaundice were used to compare the effects of DCC and ECC on neonatal jaundice as secondary outcomes: highest TsB level (days 3–5); excess value of TsB; undergoing phototherapy; and transcutaneous bilirubin levels (on days 1–4). The TsB level (days 3–5) was 12.8 mg/dL in the DCC group and 12.2 mg/dL in the ECC group, showing no significant difference (RR = 0.6, 95% CI -0.61-1.80; Table 2). Six neonates (9.1%) were over the excess value of TsB in the DCC group, compared with five (7.6%) in the ECC group (RR = 1.2, 95% CI 0.39–3.74; Table 2). Seven neonates (10.3%) in the DCC group and five (7.1%) in the ECC group underwent phototherapy, showing no significant difference (RR = 1.4, 95% CI 0.48–4.25; Table 2). Although no significant differences were evident, the DCC group tended to show higher values for all four markers of neonatal jaundice compared with the ECC group. These differences also seemed slightly wider in the per-protocol analysis and as-treated analysis (Tables 3, 4). Some TsB values were missing for days 3–5 in 6 neonates (4.3%; DCC, *n* = 2; ECC, *n* = 4); these values could not be recorded, because five neonates had been transferred to other hospitals, and one value was not recorded on the data collection sheet. For the same reason, five phototherapy values were missing (3.6%; DCC, *n* = 2; ECC, *n* = 3) (Table 2). For transcutaneous bilirubin levels on days 1–4, data could not be collected, because the neonates were transferred to other hospitals or the hospitalization was shortened by 4 days because of hospital policy or on request of the mother.

**Table 3:** Per-protocol Analysis for Transcutaneous Hb at 4 months, Neonatal Jaundice and Polycythemia

	DCC		ECC		Relative risk (95% CI) / Mean difference (95% CI)		*p* value
**Primary outcome**							
**Spectrophotometric hemoglobin (SpHb) at 4 months**							
(*n* = 127/DCC = 65,ECC = 62)	12.4	(0.8)	12.3	(0.7)	0.1	[−0.17, 0.35]	0.50
**Secondary outcome**							
**Anemia** (SpHb < 11.0 g/dl) (*n* = 126/DCC = 64,ECC = 62)	1	(1.6)	0	(0.0)	1.0	[0.95, 1.02]	1.00
**Neonatal jaundice**							
**Total serum bilirubin (TsB) (days 3-5)**) ^a^ (*n* = 122/DCC = 63, ECC = 59)	12.8	(3.5)	12.1	(3.4)	0.7	[−0.55, 1.91]	0.27
**Over the excess level of transcutaneous bilirubin (TsB)** ^b^ (*n* = 122/DCC = 63, ECC = 59)	6	(9.5)	4	(6.8)	1.4	[0.42, 4.73]	0.75
**Phototherapy** ^c^ (*n* = 123/DCC = 63, ECC = 60)	7	(11.1)	4	(6.7)	1.7	[0.51, 5.40]	0.53
**Hematocrit**							
**Hematocrit level (days 3-5) (%)** ^a^ (*n* = 121/DCC = 62, ECC = 59)	57.2	(5.3)	52.5	(5.2)	4.6	[2.74, 6.53]	< 0.01*
**High hematocrit (Hct≧65%)** (*n* = 121/DCC = 62, ECC = 59)	3	(4.6)	2	(3.0)	0.7	[0.10, 4.00]	0.68

Data are presented as means (*SD*) or *n* (%)

^a^ The highest value among blood sampling conducted during days 3-5 were used

^b^ Over the excess value are based on Standard of Imura (1985)

^c^ Two (DCC = 1, ECC = 1) received phototherapy before met to standard of phototherapy for preventively.One (ECC = 1) did not received phototherapy even over level of the standard of phototherapy

*p < .05

**Table 4:** As-treated Analysis for Transcutaneous Hb at 4 months, Neonatal Jaundice and Polycythemia

	DCC		ECC		Relative risk (95% CI) / Mean difference (95% CI)		*p* value
**Primary outcome**							
**Spectrophotometric hemoglobin (SpHb) at 4 months**							
(*n* = 137/DCC = 72, ECC = 65)	12.4	(0.8)	12.3	(0.7)	0.1	[−0.18, 0.32]	0.59
**Secondary outcome**							
**Anemia** (SpHb < 11.0 g/dl) (*n* = 137/DCC = 72, ECC = 65)	1	(1.4)	0	(0)	1.0	[0.96, 1.01]	1.00
**Neonatal jaundice**							
**Total serum bilirubin (TsB) (days 3-5)** ^a^ (*n* = 132/DCC = 70, ECC = 62)	12.9	(3.4)	12.0	(3.5)	0.9	[−0.33, 2.08]	0.15
**Over the excess level of transcutaneous bilirubin (TsB)** ^b^ (*n* = 132/DCC = 70, ECC = 62)	7	(10.0)	4	(6.5)	1.6	[0.48, 5.04]	0.54
**Phototherapy** ^c^ (*n* = 133/DCC = 70, ECC = 63)	8	(11.4)	4	(6.3)	1.8	[0.57, 5.69]	0.38
**Hematocrit**							
**Hematocrit level (days 3-5) (%)** ^a^ (*n* = 131/DCC = 69, ECC = 62)	57.0	(5.1)	52.4	(5.2)	4.6	[2.84, 6.41]	< 0.01*
**High hematocrit** (Hct≧65%) (*n* = 131/DCC = 69, ECC = 62)	3	(4.3)	2	(3.2)	0.7	[0.12, 4.54]	1.00

Data are presented as mean (*SD*) or *n* (%)

^a^ The highest value among blood sampling conducted during days 3-5 were used

^b^ Over the excess value are based on Standard of Imura (1985)

^c^ Two (DCC = 1, ECC = 1) received phototherapy before met to standard of phototherapy for preventively. One (ECC = 1) did not received phototherapy even over level of the standard of phototherapy

*p < .05

Only the hematocrit level at days 3–5 was significantly higher in the DCC group (57.0%) compared with that in the ECC group (52.6%; MD 4.4, 95% CI 2.61–6.20) (Table 2). Although no significant differences were identified, the MD in infant weight between groups was 88.5 g (95% CI -27.26-204.19 g) at birth, 30.1 g (95% CI -129.64-189.93) at 1 month, and − 3.3 g (95% CI -258.62-252.09) at 4 months (Table 2). Other secondary outcomes showed no significant differences (Table 2).

### Per-protocol and as-treated analyses

Per-protocol analysis and as-treated analysis were performed to compare the results between the two groups. The median cord clamping time was 7 s (SD = 3 s; range, 3–15 s) in the ECC group and 126 s (SD = 125 s; range, 17–622 s) in the DCC group with per-protocol analysis and 7 s (SD = 3 s; range, 3–15 s) in ECC group and 123 s (SD = 122 s; range, 17–622 s) in the DCC group with as-treated analysis. These sub-analyses did not reveal significant differences for the primary outcome of SpHb at 4 months between the intervention and control groups (Tables 3, 4). Furthermore, no significant differences in neonatal jaundice or other secondary outcomes between the groups were found. However, significant differences in the hematocrit at 3–5 days were found (Tables 3, 4).

## Discussion

### Main findings

This randomized controlled trial comparing ECC and DCC sought to explore their effects on the incidence of anemia in infancy at 4 months of age in a high-risk population of mothers and neonates with planned exclusive breastfeeding. Our results indicated that a delay in clamping the cord after more than a minute or when cord pulsation stopped had no significant effect on infant SpHb levels at 4 months compared with early clamping, in which the median clamping time was 8 s according to intention-to-treat analysis. There was no significant difference between the DCC and ECC groups in jaundice, the secondary outcome. DCC increased hematocrit levels at days 3–5 within the normal range.

### Strengths

This was the first randomized controlled trial to report timing of cord clamping for Japanese term breastfed infants. The targeted population had a high rate of exclusive breastfeeding and was considered at high risk for infant anemia as well as high risk of neonatal jaundice because of East Asian ethnicity. The results of this study may contribute to the development of clinical guidelines, specifically, guidelines considering the risks jaundice with DCC. The World Health Organization guidelines suggest that the risk of serious hyperbilirubinemia associated with DCC should be examined [23]; furthermore, Japanese guidelines highlight the need for high-quality research on the timing of cord clamping to make recommendations for clinical practice [12]. The results of this study provide strong evidence for developing recommendations in the Japanese guidelines. A further strength of this study was the high participant follow-up rate at 4 months.

### Limitations

Several limitations of this study warrant acknowledgment. First, we used SpHb to evaluate anemia, rather than hematological indices, such as ferritin or mean corpuscular volume, which are more detailed measures for assessing iron deficiency and have reportedly shown significant effects from DCC in previous studies [5, 6]. Second, although an acceptable correlation (r = 0.73) has been identified between SpHb and serum Hb [19], SpHb cannot be simply compared with serum Hb. Third, the population in this study included only breastfed, healthy, term infants with mothers who had no maternal complications; therefore, these results may not apply to mothers and neonates with complications. We choose SpHb because it has been reported to correlate with serum Hb [19] and it is a non-invasive method in neonates to collect data. In previous study, differences have been reported between ECC and DCC groups using serum Hb [21, 24], but it was not appropriate index for this study due to its invasive nature.

### Interpretation (in light of other evidence)

This study targeted mothers who were willing to exclusively breast feed and found that delay in clamping the cord after more than a minute or when cord pulsation stopped had no significant effect on infants’ SpHb level at 4 months, compared with that in the ECC group (cord clamped within 15 s). The secondary analyses: per-protocol, and as-treated analysis, did not change the outcomes. Over 80% of mothers were exclusively breastfeeding in this targeted population. The results of this study are comparable to those of previous studies in different racial groups [25, 26]. Conversely, a study in India showed significantly higher Hb concentrations in the DCC group [24]. Maternal Hb levels were lower (Hb < 10.0 g/dL), and both birth weights and Hb levels in infants were much lower than in the current study [24]. A study comparing time of cord clamping in Mexican infants found that DCC had a greater effect on body iron and stored iron in infants who were born to mothers with low ferritin levels than infants born to mothers with normal ferritin levels [6]. Anemia in pregnancy is associated with increased rates of low birth weight [27], and low birth weight infants are at high risk of anemia in infancy [28]. This study hypothesized that participants were at high risk of anemia in infancy due to being exclusively breastfed at 4 months, but the population in this study may not have been at high risk. Only one neonate showed positive results for screening for anemia, the rates of maternal anemia were average, and birth weight and growth were normal. Therefore, in addition to exclusive breastfeeding, further randomized controlled trials using DCC and ECC are required, targeting subtypes, such as populations at higher predicted risk of anemia.

For neonatal jaundice, no significant difference was evident between the DCC and ECC groups. However, the DCC group tended to have higher values for all four outcomes related to hyperbilirubinemia, compared with the ECC group. These results were similar to those of other studies in East Asian populations. Randomized controlled trials in China and Taiwan reported no significant effects on jaundice-related outcomes [7, 8]. Although not significant, MDs in transcutaneous bilirubin values of 0.5–0.6 mg/dL have been reported in DCC groups [7, 8]. In the high-risk Nepali population, due to living at high altitude, comparisons of the timing of cord clamping resulted in no significant differences for transcutaneous bilirubin values [29]. A Cochrane systematic review [9] reported that fewer infants in the ECC group required phototherapy for jaundice than in the DCC group. The possibility that neonatal jaundice is increased in the DCC group cannot be dismissed. However, we consider that the differences in total serum or transcutaneous bilirubin levels of about 0.6 mg/dL, with average values of 12.2–12.8 mg/dL, may have no large effects clinically, assuming the effects are limited. Participants of this study were low-risk women and neonates delivered at birth centers or clinics, and obstetric outcomes related to jaundice, such as prolonged labor or vacuum extraction, were few. From our results, we postulate that the danger of jaundice does not warrant recommending the practice of ECC for low-risk births at birth centers or clinics. In addition, the rate of exclusive breastfeeding is higher among women delivering at birth centers than among women who give birth in hospital [30]. While this study did not detect a positive effect on SpHb, in the DCC group, considering the negative effect of ECC on the anemia in infancy along with and the potential risks of anemia with exclusive breastfeeding, ECC may not be an appropriate practice for this population. Recommendation of timing of cord clamping may differ according to the characteristics of women and neonates.

The only values that were significantly higher with DCC than with ECC were hematocrit levels at days 3–5, with analysis according to the intention-to-treat principle, per-protocol analysis, and as-treated analysis. In all analyses, values were within the normal range in both groups (DCC = 57% vs. ECC = 52–53%), with approximately a 4.5% higher mean in the DCC group. This may be an indication of the positive effects of DCC in terms of preventing anemia in the neonatal period. These results were similar to those of previous studies that found a significantly higher hematocrit with DCC [21, 31]. One study suggested that the effects lasted 2 months [31]. In these studies, no harmful outcomes from high hematocrit-related effects were reported [21, 31]. Although no statistically significant differences were evident, the MD in infant weight was 88.5 g, and weight tended to be higher in the DCC group. The increased hematocrit in DCC may be the result of increased blood volume, as there were no differences in other baseline data related to the hematocrit. High hematocrit contributed to preventing anemia in the neonatal period but can also lead to increased bilirubin [32]. Bilirubin is produced when the erythrocyte disintegrates [32]. A higher hematocrit may be related to increased TsB values or other outcomes of neonatal jaundice in the DCC group, but any effects appeared limited. The characteristics of the index used in this study to measure anemia at 4 months may explain why no anemia was detected, despite the difference in hematocrit levels. In anemia, iron depletion starts before iron deficiency and serum ferritin decreases before Hb begins to decrease. Therefore, initial iron depletion is not reflected in Hb levels. Iron depletion may have occurred at 4 months although the initial anemia status was not measured by SpHb. Further research using an anemia index, such as serum ferritin or MCV, is needed.

Furthermore, the effects of placental transfusion related to gravity need to be taken into consideration. In this study, neonates were placed on the chest or abdomen of the mother after birth. One study explored the effects of gravity on volume of placental transfusion and found no significant difference in birth weight between introitus- and abdomen-level groups [33], but since a small, reliable study posited gravity effects for placental transfusion, the results in other populations must be interpreted with caution.

## Conclusion

In this study, we found that DCC had no significant effect on SpHb levels at 4 months compared with ECC. However, DCC increased hematocrit levels at days 3–5 within the normal range, which may be an effect in terms of preventing anemia in the newborn period. Additionally, there was no statistically significant effect of DCC on the four studied outcomes of neonatal jaundice (TsB value at 3–5 days, over the excess value of TsB, phototherapy, and transcutaneous bilirubin value at days 1–4).

To assess the effects of DCC on infant anemia more clearly, further research should be conducted in a targeted population of mothers or infants at higher risk of anemia of infancy. In addition, although there remained the possibility of an increase in the values of jaundice-related outcome in the DCC group, the clinical effects appeared limited. Further studies with a larger sample size are required to assess the effects of cord clamping on neonatal jaundice.

## Data Availability

The datasets used and/or analyzed during the current study are available from the corresponding author on reasonable request.

## Abbreviations

95% CI: 95% confidence interval
DCC: Delayed cord clamping
ECC: Early cord clamping
MD: Mean difference
RR: Risk ratio
SD: Standard deviation
SpHb: Spectrophotometric hemoglobin
TsB: Total serum bilirubin
